# Probing Solid-Binding Peptide Self-Assembly Kinetics Using a Frequency Response Cooperativity Model

**DOI:** 10.3390/biomimetics10020107

**Published:** 2025-02-12

**Authors:** Taylor Bader, Kyle Boone, Chris Johnson, Cindy L. Berrie, Candan Tamerler

**Affiliations:** 1 Bioengineering Program, University of Kansas, Lawrence, KS 66045, USA; tbader1@ku.edu; 2 Institute for Bioengineering Research, University of Kansas, Lawrence, KS 66045, USA; kyle.boone@ku.edu; 3 Department of Mechanical Engineering, University of Kansas, Lawrence, KS 66045, USA; 4 Department of Chemistry, University of Kansas, Lawrence, KS 66045, USA; cjohnson993@ku.edu (C.J.); cberrie@ku.edu (C.L.B.)

**Keywords:** adsorption, solid-binding peptides, gold-binding peptide, bio–hybrid interfaces, binding kinetics, frequency response

## Abstract

Biomolecular adsorption has great significance in medical, environmental, and technological processes. Understanding adsorption equilibrium and binding kinetics is essential for advanced process implementation. This requires identifying intrinsic determinants that predict optimal adsorption properties at bio–hybrid interfaces. Solid-binding peptides (SBPs) have targetable intrinsic properties involving peptide–peptide and peptide–solid interactions, which result in high-affinity material-selective binding. Atomic force microscopy investigations confirmed this complex interplay of multi-step peptide assemblies in a cooperative modus. Yet, most studies report adsorption properties of SBPs using non-cooperative or single-step adsorption models. Using non-cooperative kinetic models for predicting cooperative self-assembly behavior creates an oversimplified view of peptide adsorption, restricting implementing SBPs beyond their current use. To address these limitations and provide insight into surface-level events during self-assembly, a novel method, the Frequency Response Cooperativity model, was developed. This model iteratively fits adsorption data through spectral analysis of several time-dependent kinetic parameters. The model, applied to a widely used gold-binding peptide data obtained using a quartz crystal microbalance with dissipation, verified multi-step assembly. Peak deconvolution of spectral plots revealed distinct differences in the size and distribution of the kinetic rates present during adsorption across the concentrations. This approach provides new fundamental insights into the intricate dynamics of self-assembly of biomolecules on surfaces.

## 1. Introduction

Biomolecular adsorption is a critical phenomenon in biomedical and biotechnological applications [1,2,3,4]. However, these interactions are mostly referred to as nonspecific adsorption. Owing to their material-specific interactions, there has been a growing interest in solid-binding peptides enabling controllable bio–hybrid interfaces and material designs with a variety of functions used in many fields [5,6,7,8,9,10,11]. Predicting the adsorption properties under different conditions related to their application is key to their successful implementation. Yet, most biomolecular adsorption studies to date on inorganic surfaces use adsorption isotherms with non-cooperative models based on the Langmuir model or its derivatives to describe the kinetics [12,13,14]. This coarse approximation of the adsorption does not account for how biomolecules interact with each other or how adsorption changes the binding process as the surface fills according to the assumptions made in the Langmuir adsorption derivation [15,16]. In the field of Genetically Engineered Peptides for Inorganics (GEPIs) or solid-binding peptides (SBPs), a limited number of studies have partially remedied this with the use of a secondary peptide–peptide–inorganic binding constant, acknowledging the complexity of the ongoing binding events on the surface [17,18]. A large portion of studies use models that exclude cooperative interactions between the SBPs and the inorganic surfaces and the previously demonstrated assembly of the SBPs on the surface [19,20,21]. A notable exception is the recent study examining the cooperative binding of proteins with a silica-binding peptide (CAR9) to silica, modeled with a Frumkin–Fowler–Guggenheim isotherm [15].

Cooperative models have a varying exponential rate of change and, therefore, are complex enough to simultaneously describe growth kinetics as well as convergence kinetics in a single model, while non-cooperative models are limited to convergence kinetics. The binding energy landscape is dynamic for peptides adsorbing to a surface such that multiple kinetic constants are required to obtain a clear picture of semi-stable transitional formations from on-surface processes beyond the main effect approach of a single kinetic constant. Peptides self-assembling into ordered structures change the surface energy landscape progressively, as evidenced by the fact that multiple kinetic constants are necessary to reconstruct the observed adsorption profile to high precision. The field of protein kinetics has a long history of studying the cooperativity of protein–ligand binding, beginning with the change in oxygen adsorption kinetics of the hemoglobin molecule dependent on the partial pressure of carbon dioxide [22]. A degree of cooperativity was recently reported in a protein adsorption investigation for bovine serum albumin (BSA) on gold nanoparticles in addition to fluorescence lifetimes in ultrasmall gold nanoclusters [23,24,25].

While binding kinetics and affinity of the peptide to its target surface have long been modeled quantitatively, some relevant mathematical tools in physical chemistry for describing the cooperativity have yet to be applied to peptide adsorption. Analysis of kinetic equations with summed frequency responses is a tool that explicitly considers the rates at which stoichiometric reactions occur when predicting the likely compositions of complex chemical reactors. This method is known as non-linear frequency response (NFR). In 1962, Napthali and Polinski used Fourier analysis to describe the multiple phases of adsorption of hydrogen on a nickel catalyst, and explicit frequency components were attributed to different hydrogen adsorption states, including adsorption to atomic oxygen or molecular oxygen [26]. This distinction was proposed based on the unique frequency components of the adsorption process. Reyes et al. extended this type of modeling into mesoporous solids and catalysis by adjusting the response functions from those in the previous work, providing an example of a material surface serving as a molecular ligand [27]. Further studies discussed the dynamics of chemical reactors based on their frequency response characteristics [28]. To provide a standard framework for analyzing chemical reaction data, a computer-aided NFR tool (cNFR) was recently reported for chemical process design with a user interface in Excel and algorithms implemented in MATLAB to automate associated calculations [29].

Current models lack the expressiveness to include more than one effect during a long chain of adsorption events. To address this knowledge gap, there are several models with multiple events that have been applied recently in the literature [15,17,19]. However, these models still unnecessarily limit the number of resolvable events despite the continuous nature of the sensor transmitting effects happening across a range of kinetic rates. The recovery of latent information in complex, sequential data sets, such as time-varying quantities, could be beneficial to recover the latent information rather than applying a set of given kinetics. A frequency–response-based analysis can detect changes in the rate of kinetics as they happen. Within the continuous data, this type of analysis provides a better approximation of the continuous flow of effects as they happen [30].

Experimental techniques commonly used to study the binding kinetics of peptide onto inorganic surfaces include surface plasmon resonance (SPR) and quartz crystal microbalance with dissipation (QCM-D). SPR uses the change in index of refraction from a gold film to quantify the coverage of the adsorbed peptide [17]. QCM-D as an acoustic spectroscopic technique allows for real-time monitoring of the interactions between molecules and a surface through changes in resonant frequency and dissipation [17,31,32,33,34]. As molecules deposit on the sensor surface, the frequency characteristics of the crystal’s oscillations change [17,32]. The change in dissipation in response to mass deposition on the sensor surface provides insight into the viscoelastic properties of the formed molecular film on the crystal’s surface [32,33]. Over the last decade, QCM-D has been widely used to study the interactions between biomolecules and surfaces [10,17,34,35]. The high sensitivity of the method makes it valuable for characterizing the dynamic self-assembly kinetics of SBPs [10,14,17]. The technique provides related time and frequency information on dissipation along with adsorption simultaneously. Methods explicitly describing frequency dependencies can advance the knowledge gained from studies quantifying both peptide adsorption quantity shifts and peptide assembly phases, which trend in both time and frequency.

Adsorption studies on self-assembled SBPs have been expanding over a decade [21,36,37,38,39,40,41]. These peptides have gained interest for next-generation technologies where orientation and binding specificity are continued challenges, including multifunctional systems that involve bioactive components, such as enzymes, as well as bio–hybrid hierarchical materials [37,38,39,42,43,44,45,46,47,48,49]. Through dynamic, non-covalent interactions with inorganic surfaces, SBPs self-assemble on surfaces with material selective affinity [50,51,52]. This group of peptides was shown to recognize certain crystallographic facets that lead to the shaping of palladium particles [53] and provide controllable properties critical to bioelectronics [36,52,54,55]. To accelerate the translation of SBPs into applications, many peptide–material systems were studied to develop an understanding of their adsorption properties and the affinity estimates for their target solid surfaces [17,36,38,39,52,54,55,56,57,58,59]. Yet, the most common practice in peptide adsorption studies often employs non-cooperative models despite their demonstrated cooperative behaviors [19].

In this paper, we propose a Frequency Response Cooperativity (FRC) model to develop an understanding of solid-binding peptide–material interactions. Our proposed model fits time-dependent adsorption data within the frequency domain by simultaneously applying cooperative models across the frequency spectrum. Developed using the discrete Fourier transform method, akin to analyzing competing adsorption isotherms in chemical processes [60], the FRC approach increases the scope of the observable kinetic processes significantly. Our experimental focus involves monitoring the adsorption of the gold-binding peptide, AuBP1, onto gold surfaces using atomic force microscopy (AFM) and QCM-D. AuBP1 was selected due to its well-characterized self-assembly behavior, facilitating comparisons with previously published values [14,37,38,52,57,61,62]. Lower ionic strength conditions were selected to decelerate peptide motions, allowing for detailed observations of adsorption processes. Our system in this work enables the investigation of peptide behavior under conditions favoring thermodynamic order, emphasizing low concentrations and lower ionic strength compared to prior studies [57,63]. The deconvolution of the kinetic profiles is associated with each concentration, highlighting changes in the adsorption energy landscape during the distinct kinetic processes happening on the surface and providing a more nuanced description of peptide self-assembly on gold surfaces.

## 2. Materials and Methods

### 2.1. Materials

Materials for peptide synthesis included N-methyl morpholine (NMM), Wang amide resin, Fmoc-resin, Fmoc-amino acid building blocks, piperidine, and 2-(1H-benzotriazole1-yl)1,1,3,3-tetramethyluranium hexafluorophosphate (HBTU), which were purchased from AAPTec LLC (Louisville, KY, USA). N, N-dimethylformamide (DMF, 99.8%), trifluoroacetic acid (TFA, 99%), triispropyl silane (TIS, 98%), thioanisole (99%), and diethyl ether (99%) were obtained from Sigma-Aldrich (St. Louis, MO, USA). 1,2-ethanedithiol (95%) and 1,2-dimercaptoethan (EDT, 95%) were obtained from Acros Organics (Fair Lawn, NJ, USA). Phenol (89%), potassium phosphate monobasic (KH_2_PO_4_, 99.9%), potassium phosphate dibasic anhydrous (K_2_HPO_4_, 99.4%), and potassium chloride (KCl, 99.7%) were obtained from Fisher Scientific (Fair Lawn, NJ, USA). All chemicals were used without further purification. Gold-coated QCM-D sensors (QXS 301) with a 5 MHz fundamental frequency and a diameter of 14 mm were obtained from Nanoscience Instruments (Phoenix, AZ, USA).

### 2.2. Peptide Synthesis

The AuBP1 peptide was synthesized on the AAPTec FocusXC synthesizer (AAPTec, Louisville, KY, USA) using a standard Fmoc solid-phase peptide synthesis protocol, as described previously [64,65]. Following synthesis, peptides were cleaved from the Wang resin, and protecting groups were removed using a cleavage cocktail (81.5% TFA, 1.0% TIS, 5.0% DI water, 2.5% EDT, 5.0% thioanisole, 5.0% phenol). Peptides were incubated in the cleavage cocktail on a rotator for 3 h, precipitated in cold ether, and then lyophilized to obtain the crude peptide. Purification of the crude peptide was performed on a semi-preparative reversed-phase high-pressure liquid chromatography (HPLC) Waters system, equipped with a Waters 600 controller, a Waters 2487 Dual Absorbance Detector, and a 10 µm C-18 silica Luna column (250 × 10 mm Phenomenex Inc., Torrance, CA, USA). Mobile phases were composed of phase A (94.5% HPLC-grade water, 5.0% acetonitrile, 0.1% TFA) and phase B (100% acetonitrile). Lyophilized peptide was dissolved in 4 mL of phase A and 1 mL of phase B and purified at 0.5% phase B × min^−1^ on a linear gradient (5–85% phase B), with detection at 254 nm. Purified peptides were then lyophilized and stored at −20 °C.

A sample of lyophilized purified peptide was sent to the Mass Spectrometry and Analytical Proteomics Laboratory at the University of Kansas for Electrospray Ionization Mass Spectrometry confirmation of molar mass. The results indicated that the dominant species in the sample had a molar mass of 1454.7, which matches the expected molar mass of the peptide.

### 2.3. AFM Studies

AFM samples were prepared using Au(111) purchased from Phasis and with AuBP1 dissolved in a potassium phosphate buffer of pH 7.4 at 1 mM with 10 mM KCl to mimic biological conditions. The samples were cleaned with Millipore water followed by a buffer rinse, and then a 25 µL droplet of the peptide buffer solution was adsorbed onto the surface for one hour at varying concentrations (1 fM, 1 nM, 100 nM, 10 µM). The peptide solution was then washed from the surface with Millipore water to remove any loosely bound peptide and dried with a stream of nitrogen before imaging.

### 2.4. QCM-D Studies

Q-Sense E4-Auto Quartz crystal microbalance with dissipation (QCM-D) (Biolin Scientific) was utilized to obtain the peptide’s experimental adsorption behavior. Gold-coated quartz QCM sensors with a fundamental frequency of 5 MHz and a diameter of 14 mm were obtained from Nanoscience Instruments (Phoenix, AZ, USA). Gold sensors were mounted in standard flow modules, and the system was filled with a 1 mM phosphate buffer solution (3:1 K_2_HPO_4_:KH_2_PO_4_) containing 10 mM KCl. Once a stable baseline was achieved, peptide solution was introduced to the system at varying concentrations (0.05, 0.075, 0.1, 0.15, 0.175, 0.25, 0.5, 0.75µM). The frequency shifts of the crystal following the addition of peptide to the system were then monitored continuously for a defined length of time to allow for the self-assembly of peptides on the gold sensor’s surface. Each experimental run obtained frequency and dissipation data from three individual sensors. Experimental data were collected for at least three separate runs, resulting in at least 9 experimental adsorption curves for each concentration. Each collected run was evaluated with respect to its corresponding dissipation data. Any runs that had steady dissipation over the course of the run and/or a dissipation change of less than 0.5 × 10^−6^ (unitless) were kept in the dataset. Any runs that disobeyed this assumption were eliminated. This steady dissipation assumption was made to distinguish true experimental data from data that exhibited an instrumental drift [33]. All of the runs that passed this dissipation filter are individually represented by concentration in Figures S1–S8. From this dataset, a representative curve was selected for each concentration for further analysis. For each individual run, its root-mean-square deviation (RMSD) from the average curve representing that concentration was calculated. The run with the minimum RMSD value was selected to represent that concentration in further analyses.

For each concentration, the frequency data from the 5th, 7th, and 9th overtones were averaged to create the experimental adsorption curve. Experimental adsorption curves were then plotted in Excel by plotting the negative change in frequency over time.

### 2.5. Frequency Response Cooperativity Model

Analyzing the frequency response in an adsorption system represents both the time-dependent changes (when processes occur) and the frequency-dependent changes (how fast processes occur). For processes that have a sudden beginning, such as the presence of peptide in a solution following an injection, a non-cooperative model, such as the Langmuir model (A × $exp(-k_{obs}$ × t)), is suitable for describing the convergence behavior of the process. But, for processes that have gradual beginnings, such as nucleation-like processes of assembly, the Langmuir model cannot capture the growth phase of the curve. Assembly processes also tend to have convergent behavior at infinity, so curves that incorporate both behaviors are suitable for modeling both the peptide adsorbing to the surface and its self-assembly. One example of such a curve is the sigmoidal curve

(1) $$∆f_{\infty ,i}\;\times \left(\frac{1}{1+exp\left(-k_{obs}\;\times (t+∆t_{i}\right)}\right)+∆f_{vertical\;shift},$$

Figure S9A shows Langmuir models shifted in time, showing its lack of flexibility in showing small changes at the beginning. Figure S9B shows the sigmoidal curve with changes in its free parameters and its flexibility to describe growth behavior as well as convergent behavior.

We identify four free parameters for the cooperative models, as represented in Equation (1):

1. $∆f_{\infty ,i}:$ This scaling factor of the hyperbolic term describes the additive contribution of the $i^{th}$ process.
2. $k_{obs,\;i}:$ The hyperbolic term multiplication constant of *k_obs_* shifts the sigmoidal curve of the $i^{th}$ process horizontally. This term is both related to the time at which the process happens at maximum velocity and the time at which half of the process shift has accumulated.
3. $∆t_{i}$: This is the time shift for the point of inflection of the sigmoid curve for the $i^{th}$ process. At this time, the curve both has the maximum rate of change and is at one-half of the final sensor frequency shift change.
4. $∆f_{vertical\;shift}:$ A vertical shift of the $i^{th}$ process curve below the horizontal axis. Our physical interpretation is that this shift fits the time of the limit of deflection, the amount of elapsed time prior to the deflection shift in sensor frequency. The initial value for this shift in curve fitting begins at zero. See Figure S10 for the limit of deflection (LOD) as a function of frequency in our adsorption measurements. The higher LOD values show when the emergence of the change in sensor frequency is more sudden and when less gradual growth kinetics can be observed.

Our method differs from a direct application of the discrete Fourier transform in that our results are not in the weights of sinusoidal functions in the form of Equation (2) where *j* is $\sqrt{-1}$; rather, our method finds summations of cooperative sigmoidal curves in the form of Equation (3).

(2) $$∆f_{t}=\sum_{i}^{n}∆f_{\infty ,\;i}\times e^{-k_{obs}\;\left(t-∆t_{i}\right)j},$$

(3) $$∆f_{t}=\sum_{i}^{n}∆f_{\infty ,\;i}\times \left(\frac{1}{1+e^{-k_{obs,\;i}\times \;\left(t-∆t_{i}\right)}}\right)-∆f_{vertical\;shift},$$

### 2.6. Peak Deconvolution

CasaXPS (Version 2.3.26) was used to deconvolve each of the kinetic spectra obtained using the FRC model. Values saved in the MATLAB code under “log_k_obs” and “curve_weight” were saved as X and Y values, respectively, in the same text file. This text file was converted to a Vamas file using CasaXPS. Analysis was performed under the quantification function. One region was created per datafile with a “W Tougaard” BG type, an average width of 1, and a standard offset of 0. Individual components were added and fit to the overall model until the optimal deconvolution was obtained.

## 3. Results and Discussion

### 3.1. Peptide Assembly at Low Concentrations

**Figure 1:**
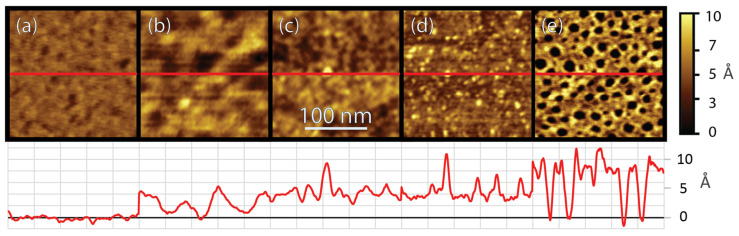
AFM height images, 250 × 250 nm^2^, with a false color scale of 10 Å of various concentrations absorbed for one hour, with cross-sections through the middle shown in red directly beneath the images, with a scale from 0 to 10 Å (adjusted so that the lowest areas have a height of 0); (**a**) bare Au(111), (**b**) 1 fM AuBP1, (**c**) 1 nM AuBP1, (**d**) 100 nM (**e**) 10 µM AuBP1. (The total height for the cross-section of (**c**,**d**) has been approximated based on completion of the initial layer, and the height shown should represent the height above the bare gold).

The progression of peptide film assembly was observed as a function of gold-binding peptide (AuBP1) concentration and the complexity of the peptide interactions, which were prominent over the course of adsorption. Figure 1 shows atomic force microscopy (AFM) images of the development of the peptide film as a function of peptide concentration in the solution used for adsorption.

At one hour of adsorption time, the surface has reached near equilibrium coverage and structure. Figure 1a shows a bare Au(111) surface with little variation across the grain. After adsorption of 1 fM AuBP1 (Figure 1b), the surface shows isolated peptide adsorption on the surface, with some aggregation and clustering of the peptide. Upon adsorption from a 1 nM AuBP1 solution (Figure 1c), a nearly complete peptide film is formed on the surface, with a height of approximately 4 Å. At 100 nM (Figure 1d), a completed initial peptide layer with multiple raised peptide features appearing as protrusions above the initial peptide film is shown. The last concentration, 10 µM (Figure 1e), shows multiple ~1 nm deep holes in the peptide film, which is significantly different compared to the lower concentrations, presumably due to distinct assembly pathways based on the differences in peptide availability and binding energy landscapes on the surface under these conditions.

The observation of such clustering, rearrangement, and reorganization on the surface at different concentrations is not consistent with a simple Langmuir adsorption model and suggests a complex interplay between peptide–gold and peptide–peptide interactions driving such aggregation and rearrangement. Also of significance is the very large coverage of peptides on the surface, even upon adsorption from peptide solutions at concentrations as low as 1 fM. This coverage deviates significantly from the coverage predicted using the Langmuir model and previously published kinetics parameters, which predict near zero equilibrium coverage at this very low concentration [66].

In addition, the evolution of the peptide film as a function of time shows similar, initially isolated adsorption, aggregation, and filling, followed by reorganization of the peptide film into higher-ordered structures. Observation of these peptide–surface, peptide–peptide, and peptide–peptide–surface interactions reveals that the process of peptide assembly on surfaces is far more complex than what has previously been characterized in the literature. This complexity, though, has been hinted at in past work using AFM to demonstrate the complex, multi-step assembly of peptides on gold surfaces as a function of both concentration and time [19].

The complex interplay of solid-binding peptide–surface interactions leading to self-assembled bio–hybrid systems indicates a deviation from the classical pathway in self-assembly processes. This interplay agrees with recent reports on the self-assembly of biomolecules and colloidal particles. Self-assembly of colloidal particles was reported to follow a two-step mechanism that includes disordered and dense cluster formation on the surface prior to developing organized phase formation [67,68]. Once surface density of the disordered clusters has reached a threshold value, the adsorbed molecules then undergo a phase transformation, reorienting on the surface to achieve the final organized phase formation. This “threshold value” is also reported to be dependent on concentration, referred to in these studies as surface density or supersaturation in the system [67,68,69]. Both our AFM and FRC model analyses support the non-classical self-assembly pathways demonstrated by the formation of peptide clusters through a concentration-dependent mechanism. As surface diffusion and reorientation continue in these regions, peptides achieve self-assembled structures, leading to robust high-affinity bio–hybrid interfaces.

Although these AFM results provide only a snapshot of what AuBP1 self-assembly looks like at discrete time points in specific locations, they provide complimentary information about the spatial layout of the individual features present during the self-assembly process. Combining these results with the QCM-D adsorption spectra adds information about the rate and quantity at which these formations begin to appear on the surface in the larger view of trends across many locations.

### 3.2. Experimental Evaluation of Peptide Adsorption over Time

**Figure 2:**
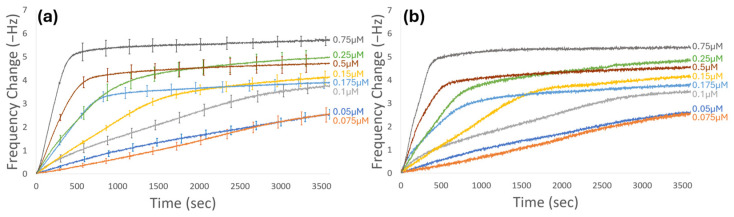
Gold-binding peptide AuBP1 (WAGAKRLVLRRE) concentration-dependent adsorption curves using low ionic strength buffer. (**a**) Averaged frequency–response curves for the adsorption of AuBP1 on a gold-coated QCM-D sensor for all runs of all concentrations. (**b**) Representative frequency response curves for each concentration used for further analysis. Curves represent the average of the 5th, 7th, and 9th harmonics for a 5 MHz sensor.

To quantify AuBP1 adsorption on gold surfaces, a continuous flow quartz crystal microbalance with dissipation (QCM-D) system was used to observe mass deposition on the sensor surface as a change in frequency. In this system, concentrations ranging from 0.05 to 0.75 µM of AuBP1 were run. The raw change in frequency as a response to the adsorption of the peptide onto the electrode surface was plotted against time. Figure 2a represents the average of at least three adsorption curves for each concentration, while Figure 2b shows the representative runs of each concentration selected for further analysis. These curves represent the averaged response of the 5th, 7th, and 9th overtones. The adsorption curves representing the selected runs for each concentration are provided in the Supplementary Materials (Figures S1–S8). In Figure 2, it is apparent that there are two distinct regimes of adsorption behavior that are concentration dependent. It appears that from 0.05 to 0.1 µM, adsorption proceeds relatively slowly, with the concentration-dependent arrival of the peptide onto the surface being the dominant process for the changing kinetics in the initial portion of the experimental run time. Consequently, the dominant process changes kinetically during adsorption. The assembly of absorbed peptides slowly changes the adsorption energy surface compared to the clean sensor. By 3600 s, adsorption curves for these concentrations seem to reach yielding points, representing shifts in the dominant kinetic process. At concentrations starting from 0.15 µM up to 0.25 µM, we see the emergence of a distinct initial slope, indicating that peptides begin to adsorb to the gold surface at a faster rate than what is seen at the lower concentrations. When reaching higher concentrations, between 0.25 and 0.75 µM, we see distinctly steeper initial slopes that indicate near-instantaneous binding to the surface, especially at 0.75 µM. Because increases in peptide solution concentration do not always result in an increase in the frequency shift at equilibrium, cooperativity, which violates the Langmuir model’s assumptions, must be present.

The concentration of peptide solutions greatly affects the adsorption and self-assembly behavior of the peptides to their target inorganic surface. It has previously been demonstrated that at low solution concentrations, neighboring adsorption sites to adsorbed peptides remain open for longer periods of time, allowing the adsorbed peptide time to change its conformation on the surface, finding the most thermodynamically favorable orientation [63]. Because the folded state of the adsorbed peptides is more likely to be well-ordered, the self-organization process is likely to achieve larger detectable areal masses and network formation. As the concentration of the peptide solution increases, these neighboring sites are filled rapidly, not allowing adsorbed peptides sufficient time to reorganize and find their thermodynamically favorable conformation [70]. From this understanding, we can say that at low concentrations, peptide adsorption and reorganization are under thermodynamic control, while, for higher concentrations, adsorption is under kinetic control. With greater surface mobility and time for thermodynamic reorganization, there is greater likelihood of peptide self-assembly into structures with greater mass and features, like semi-crystalline formation, at low concentrations. This is also why we see a distinctly different picture of self-assembly formation as the concentration of our peptide solution increases above a boundary concentration range. This could likely explain why a non-linear exponential relationship between peptide solution concentration and equilibrium frequency as the elapsed time increases is seen in Figure 2. We observe that the initial fits of the curves depend predominately on the peptide solution concentration for the kinetic adsorption rates but that the convergent frequency shift has a more complex relationship with the peptide solution’s concentration than a linear exponential fit.

An alternative explanation or additional consideration for this concentration-dependent behavior observed in the QCM-D frequency is that peptide–peptide interactions may trigger aggregation in solution, especially at higher concentrations. Peptide aggregates form in solution when the concentration exceeds a critical value [71,72]. The QCM detects and records frequency changes based on the changes in mass deposition on the sensor surface. These changes will be affected by a series of events, including peptide–surface interactions, interactions and deposition of salt ions, and entrapment of water molecules. However, solid-binding peptides are expected to replace these non-specific interactions as they go through cluster formation, reorientation, and reorganization while they achieve their self-assembled structures.

### 3.3. Effect of Ionic Conditions on Peptide Adsorption

To evaluate the kinetics of AuBP1 at low ionic strength to the gold surface through a traditional cooperative model, we fit the A.V. Hill equation model for the initial kinetics of our concentration-dependent frequency data. Using a natural log plot, we derived a linear equation representing the fit of the Hill equation to the adsorption data. The full equation and fit can be found in Equation (S1) and Figure S11. It was determined that the n value, or the cooperativity constant, for our system is 0.96, which indicates that there is negative cooperativity in the system. Negative cooperativity indicates that as peptides bind to the gold surface, the rate of subsequent peptides adsorbing to the surface decreases [73]. This rate decrease supports the claim that the energy landscape of peptide surface adsorption is dynamic and changes as peptides adsorb and assemble on the surface.

The A.V. Hill equation fit also allowed for the calculation of the kinetic equilibrium constant (K_eq_) and free energy of binding for AuBP1 in our low-concentration, low-ionic-strength system. These values, as well as those previously published for AuBP1, can be found in Table 1. When comparing our free energy of binding estimate for AuBP1 on gold surfaces, we note that the low-ionic-strength conditions used in our experiments lead to a small loss (about +0.6 kcal/mol) in free energy compared to the previously published values. The tested concentrations of K^+^ and Cl^−^ ions within the phosphate buffer increase the free energy (less favorable) compared to higher salt concentrations previously tested. This observation is evidence that the change in ionic concentration facilitates the adsorption of the peptide onto the gold surface. This salt concentration dependence may be consistently present for metal-binding peptides, which often rely on surface charges to specifically adsorb to metal surfaces [8,9,10,56]. The dependence might be due to the increased number of dissolved charges, supporting more transition states to stable adsorption conformations. Our results are comparable in free energy for the cooperative model used to the three previous non-cooperative models, indicating similar outcomes where the traditional models and the cooperative model overlap. However, the estimate of the cooperativity models helps to describe the relative magnitudes of the peptide–peptide interactions compared to the peptide–surface interaction and provides knowledge on the multitude of surface events taking place. This information is explicit using the Hill model, but it remains latent when using non-cooperative models.

### 3.4. Cooperativity-Based Modeling of Peptide Adsorption

Often, adsorption curves, like the ones obtained using QCM-D shown in Figure 2, are fit to a Langmuir isotherm to calculate the apparent kinetic rate constants for each concentration and, subsequently, kinetic constants for that overall peptide. However, it is known that there are several assumptions of the Langmuir model that our system does not obey, including monolayer formation and no lateral interactions between molecules [15,74,75,76,77]. Our group has previously expanded on the Langmuir isotherm to better fit the adsorption behavior that is being seen in peptides by developing the biexponential Langmuir model, which uses two distinct Langmuir isotherms to individually model the self-assembly regimes that were observed [17,18]. However, because many of our adsorption curves exhibit sigmoidal profiles, the interactions between peptides seem to influence the adsorption and self-assembly rearrangement in our system. This led us to investigate the use of alternative adsorption kinetic isotherms that would consider the interactions between peptides, unlike the Langmuir model. Recently, Hellner et al. used a cooperative model to probe the adsorption kinetics of the Cas9 protein tagged with green fluorescent protein (GFP) [15]. Their short peptide sequence, even without the GFP tag, showed sigmoidal adsorption profiles, indicating that their system exhibited some level of cooperativity. This change from the Langmuir to a cooperative model allowed the authors to accurately capture the adsorption kinetics of their protein. The authors then adapted a Frumkin–Fowler–Guggenheim isotherm to define the rate law expressions for their protein system, which considered the lateral interactions between peptides. Because our system has demonstrated cooperativity, as quantitatively shown in Equation (S1), we further explore its non-Langmuir characteristics.

To further investigate the balance of kinetic and thermodynamic processes occurring during adsorption, we developed a method that adapts non-linear frequency response (NFR) for series of cooperative adsorption isotherms, which can track trends across controlled variables. We call this method Frequency Response Cooperativity (FRC). We apply the same frequency response relationships for adsorption isotherms as NFR applies for multiple-step chemical reactions. We build on the current NFR-related tools by filtering likely frequency response models by fitting the collected data within computational resource limits. An important capability of the FRC method is building likely models given the adsorption isotherm data collected.

**Figure 3:**
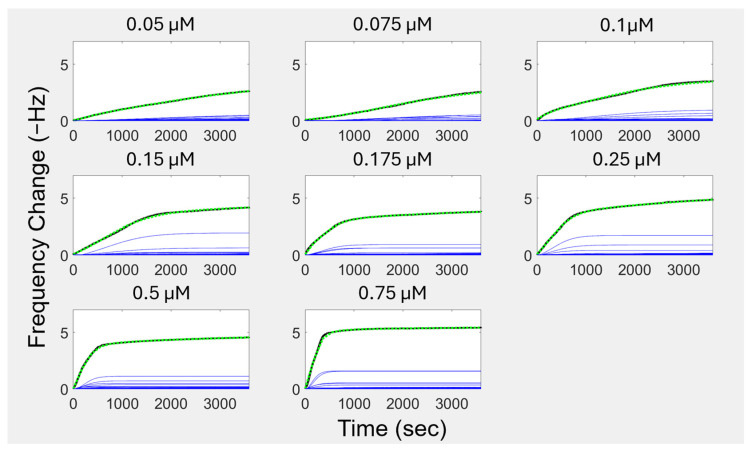
Frequency Response Cooperativity (FRC) isotherms and their components for QCM by peptide concentrations. The green curves are the FRC isotherms, reconstructing the experimental QCM data (black curves). The FRC isotherms are the sums of their cooperative components in blue. The cooperative components have growth phases and convergence phases.

For the current study, we considered a range of time on the order of an hour, taking measurements about once every second. We discretized our search over the continuous range of kinetic constants by spacing the frequency representation into 50 components, which is user definable. Each section has one cooperative curve, which is scaled by an initial rough curve-fitting procedure, followed by non-linear optimization in MATLAB (Version R2023B) with the target-reflecting procedure. The scaling of the curves acts as a logical switch, allowing for frequencies that have explicit model components but no applicable contribution to the observed data to be scaled to near zero values. The second optimization step considers when components reach their maximum rate, as described in Section 2.5, as the third of four free parameters. We initially set $∆t_{i}$ to be related to the progression of kinetic constants between user-defined limits. In the sigmoidal curve shape, the maximum rate occurs when the curve transitions from its growth phase to its convergence phase. However, the maximum amount for the curve does not occur at the maximum rate; it is approached as the time elapsed approaches infinity. The same behavior is seen in the Langmuir model, which has its maximum rate occur at an elapsed time equal to zero. The overall FRC models for each representative QCM curve are shown in Figure 3 as the dashed green curve. The QCM adsorption data curves are in black, and the series components of each of the FRC models are in blue.

**Figure 4:**
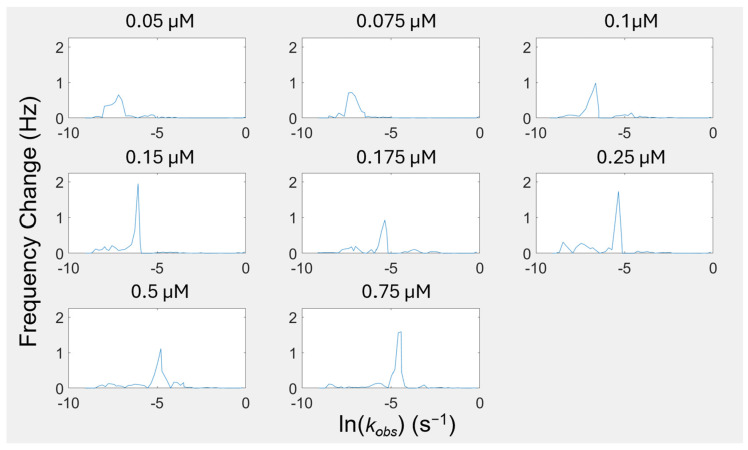
Spectral distribution of component scaling factors for apparent kinetic constants (*k_obs_*) for FRC components across AuBP1 concentrations. Concentrations of 0.05–0.1 μM are solely driven by slow kinetics. Concentrations of 0.15–0.175 μM are driven by both slow and fast kinetics. Concentrations of 0.25–0.75 μM are mainly driven by fast kinetics.

To describe the frequency response of the components of these curves in Figure 3, we constructed a component distribution plot for each curve, as seen in Figure 4. Instead of having a single estimate *k_obs_* for each experiment, we have spectrally distributed estimates, which resolve into distinct frequency bands of sensor frequency change contributions. With the *k_obs_* distribution, we can visualize the differentiation of thermodynamically driven processes and kinetically driven processes. The slowest regime includes concentrations of 0.05–0.1 μM. This regime is solely thermodynamically driven. The second regime is a transition phase. Concentrations of 0.15–0.175 μM are driven by both thermodynamic and kinetic processes, with the proportion of kinetic drive increasing with concentration. For the final third regime, concentrations of 0.25–0.75 μM are almost exclusively kinetically driven. With the visibility of shifting kinetic regimes provided by this FRC modeling technique, the tunability of such regimes is now a more promising path for engineering self-assembled systems.

**Figure 5:**
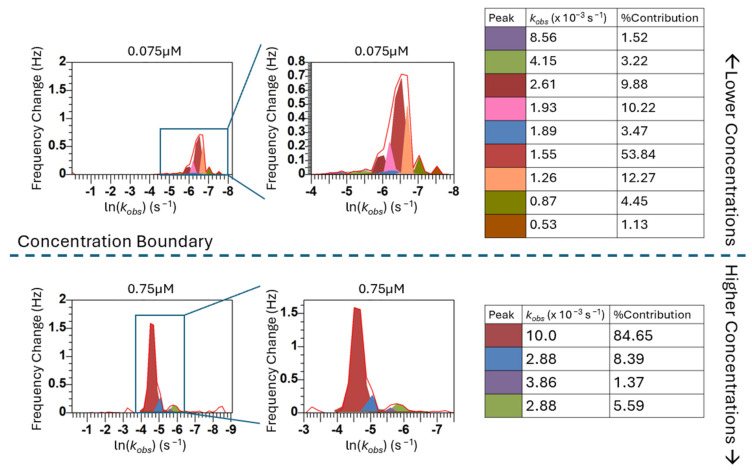
Peak deconvolution for representative concentrations.

Within the kinetic regimes identified in Figure 4, the peaks indicate that there are many self-assembly events occurring throughout the adsorption process at distinct rates (*k_obs_* values). Even when examining the largest peak, which we are defining as the “main surface assembly event” because it has the largest contribution to the overall model fit, we are seeing what appear to be overlapping and asymmetric peaks, indicating that deconvolution of the peaks is necessary to understand the more fundamental processes this peak describes. By deconvolving the peaks, we can separate these “main surface events” into individual components to uncover further information about the rates of each of these events. Figure 5 shows the deconvolution of four representative concentrations. The deconvolution of each of the concentrations used in this study can be found in Figure S12.

When we are examining the lower concentrations (0.05–0.1 µM), the individual peaks from the deconvolution are likely representative of different island formations on the surface that have discernable kinetics that are thermodynamically driven. At higher concentrations (0.25–0.75 µM), the number of peptides assembling at the surface at each time phase is getting larger. While the initial mass deposition follows linear exponential behavior (a singular *k_obs_* value) to a rough approximation, the lack of concentration dependence on equilibrium adsorption demonstrates that cooperativity is still an important effect. We are extracting more information from our adsorption phenomena than the initial kinetics. The heterogeneous kinetics of this assembly are resolvable using the FRC method in contrast to traditional methods.

We now can begin to understand the different speeds of assembly processes under different concentrations through FRC analysis. The mechanistic descriptions of advanced assembly systems with phase transformations, including concentration thresholds and reorientation processes, can now be parsed using probabilistic tools into kinetic pathways [69]. The assembly process is a function of the distribution of the kinetic processes that form stable structures on the surface. Therefore, while spectra for different biomolecule concentrations have overlapping kinetic components, the assembled result may be highly sensitive to small concentration changes. Understanding where these boundary conditions exist is a fundamental challenge to developing nanoscale technologies with this approach, which is addressed by our model.

## 4. Conclusions

Understanding the adsorption properties of solid-binding peptides on materials is essential for advancing their utilization in diverse applications from medicine to environmental science and technology. As solid-binding peptides continue to advance and find broader applications, there is a growing need for models that capture the complex cooperative interactions involved in their adsorption process, beyond the non-cooperative approaches previously studied. In response to the complex behaviors observed in solid-binding peptides, this study introduces a Frequency Response Cooperativity (FRC) model that deciphers distinct kinetic processes, offering deeper insights into peptide self-assembly dynamics on material surfaces. This study explored the distribution of the apparent kinetic rate constants of a widely used gold-binding peptide, AuBP1, on gold surfaces using quartz crystal microbalance with dissipation (QCM-D) across a range of concentrations. By implementing a new FRC model, we identified trends of the distinct kinetic processes underlying peptide assembly on the QCM sensor, leveraging latent frequency data in a manner akin to non-linear frequency response (NFR) analysis of chemical reactions. This approach enabled rough estimates of subcomponent processes involved in self-assembly structures at lower peptide concentrations. Our results reveal that at lower concentrations, peptides exhibit sufficient spatial freedom to unfold and explore various conformations on the gold surface, leading to a more diverse array of kinetic processes. This behavior contrasts with higher concentrations, where peptides seek thermodynamically optimal conformations more rapidly and less variably. This progression of states depicted in AFM images showcased the evolution of adsorption energy landscapes from bare surface or monolayer peptide interactions into multi-layered peptide assemblies with multiple assembly pathways influenced by local assembly conditions. Component distribution plots show that distinctive self-assembly features require time and spatial distance to resolve, resulting in varied sizes and shapes. The interdependence between local assembly conditions and initial conditions complicates efforts to solve complex assembly challenges. By incorporating frequency-dependent information, our method offers a more comprehensive representation of this intricate interdependence. This work lays a critical foundation for advancing models of biomolecular self-assembly, providing a framework to explore the intricate dynamics of adsorption at solid interfaces. By refining our understanding of peptide interactions and cooperative binding kinetics, this approach has the potential to inform future studies, ultimately guiding the design of more predictable and tunable nanostructures with broad applications in biotechnology and materials science.

## Figures and Tables

**Table 1:** Free-energy parameters for AuBP1 studies on the gold surface.

	K_eq_ (M^−1^)	∆G_B.E._ (kcal/mol)	Concentrations	Ionic Strength	Model
Current Study	1.06 ± 0.75 × 10^6^	−8.23 ± 0.16	0.05–0.75 µM	1 mM phosphate buffer with 10 mM KCl	Hill equation
Hnilova, et al., 2008 [57]	3.24 ± 1.31 × 10^6^	−8.9 ± 0.2	0.23–2 µM	10 mM phosphate buffer with 100 mM KCl	Langmuir model
Hughes, et al., 2021 [61]	Not reported	−9.73 ± 0.5	2–10 µM	Not reported	Langmuir model
Palafox-Hernandez, et al., 2014 [62]	Not reported	−8.98 ± 0.22	2–10 µM	Not reported	Langmuir model
Tang, et al., 2013 [14]	4.0 × 10^6^	−8.99 ± 0.22	2–10 µM	Not reported	Langmuir model

## Data Availability

The original contributions presented in this study are included in the article/Supplementary Material. Further inquiries can be directed to the corresponding author.

## Supplementary Materials

The following supporting information can be downloaded at https://www.mdpi.com/article/10.3390/biomimetics10020107/s1, Figure S1: All frequency and dissipation data collected for 0.05 µM AuBP1; Figure S2: All frequency and dissipation data collected for 0.075 µM; Figure S3: All frequency and dissipation data collected for 0.1 µM AuBP1; Figure S4: All frequency and dissipation data collected for 0.15 µM AuBP1; Figure S5: All frequency and dissipation data collected for 0.175 µM AuBP1; Figure S6: All frequency and dissipation data collected for 0.25 µM AuBP1; Figure S7: All frequency and dissipation data collected for 0.5 µM AuBP1; Figure S8: All frequency and dissipation data collected for 0.75 µM AuBP1; Figure S9: Spectral isotherm descriptions; Figure S10: Limit of deflection (LOD) as a function of frequency in adsorption measurements; Equation (S1): Derived Hill equation for the initial kinetics of AuBP1 using QCM-D data; Figure S11: Hill equation fit for initial QCM-D frequency data; Figure S12: Peak deconvolution for all concentrations of AuBP1.
