# Supplementary material for: Probing Solid-Binding Peptide Self-Assembly Kinetics Using a Frequency Response Cooperativity Model
Source: Biomimetics (Basel). 2025 Feb 12;10(2):107. doi: 10.3390/biomimetics10020107 (PMC11853711; doi:10.3390/biomimetics10020107)
Supplement: Supplementary file 1 [file biomimetics-10-00107-s001.zip › Supplemental Materials for FRC Paper_vFinal.docx]

**Supplemental Materials**

Probing Solid-Binding Peptide Self-Assembly Kinetics by Frequency Response Cooperativity Model

Taylor Bader ^1,2,†^, Kyle Boone ^2,3,†^, Chris Johnson^4^, Cindy L. Berrie^4^, and Candan Tamerler ^1,2,3,^*

^1^ Bioengineering Program, University of Kansas, Lawrence, KS, 66045, USA

^2^ Institute for Bioengineering Research, University of Kansas, Lawrence, KS, 66045, USA

^3^ Department of Mechanical Engineering, University of Kansas, Lawrence, KS, USA

^4^ Department of Chemistry, University of Kansas, Lawrence, KS, 66045, USA

***** Correspondence: [ctamerler@ku.edu](mailto:ctamerler@ku.edu)

**^†^** These authors contributed equally to this work

**
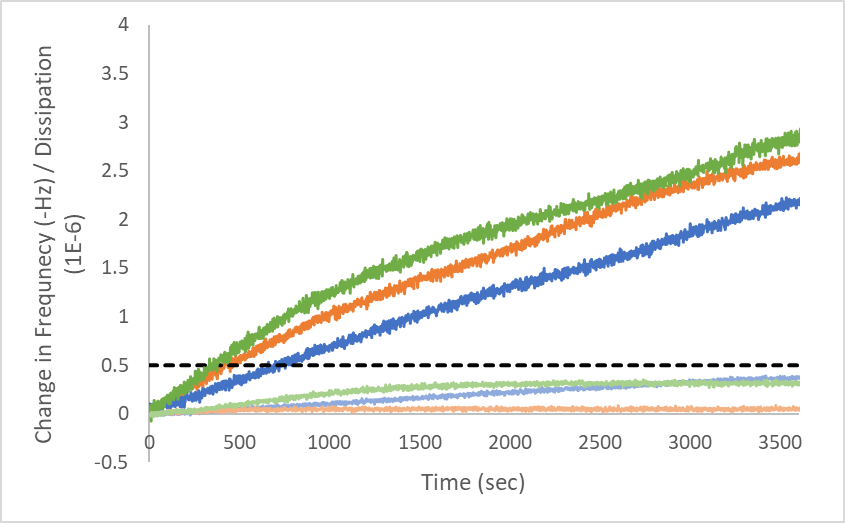
**

**Supplemental Figure 1.** All frequency and dissipation data collected for 0.05 µM AuBP1. The black dotted line represents the limit of dissipation change that we allowed for this work. The distinct colors represent different experimental runs, while the faded colors represent the dissipation measurement for that corresponding run. The orange curve is the representative curve selected for further analysis.

**
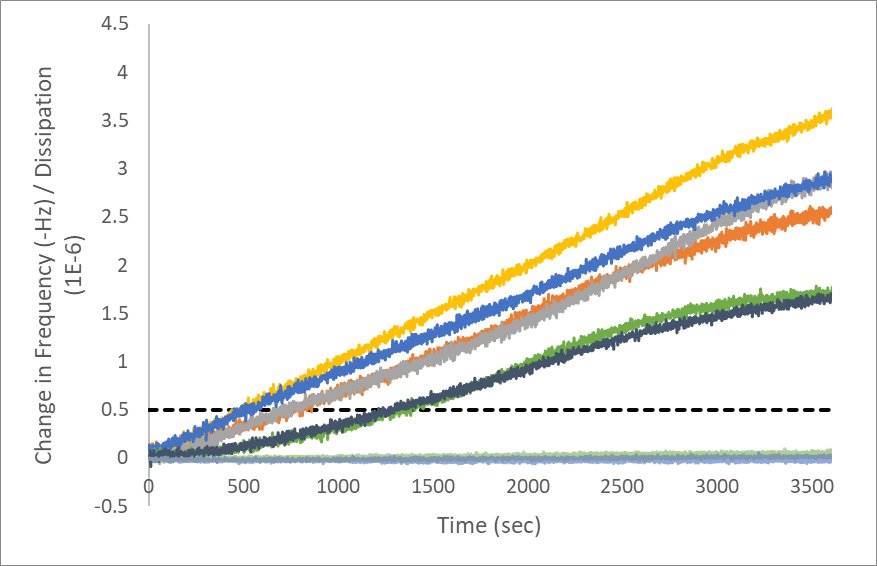
**

**Supplemental Figure 2.** All frequency and dissipation data collected for 0.075 µM AuBP1. The black dotted line represents the limit of dissipation change that we allowed for this work. The distinct colors represent different experimental runs, while the faded colors represent the dissipation measurement for that corresponding run. The orange curve is the representative curve selected for further analysis.


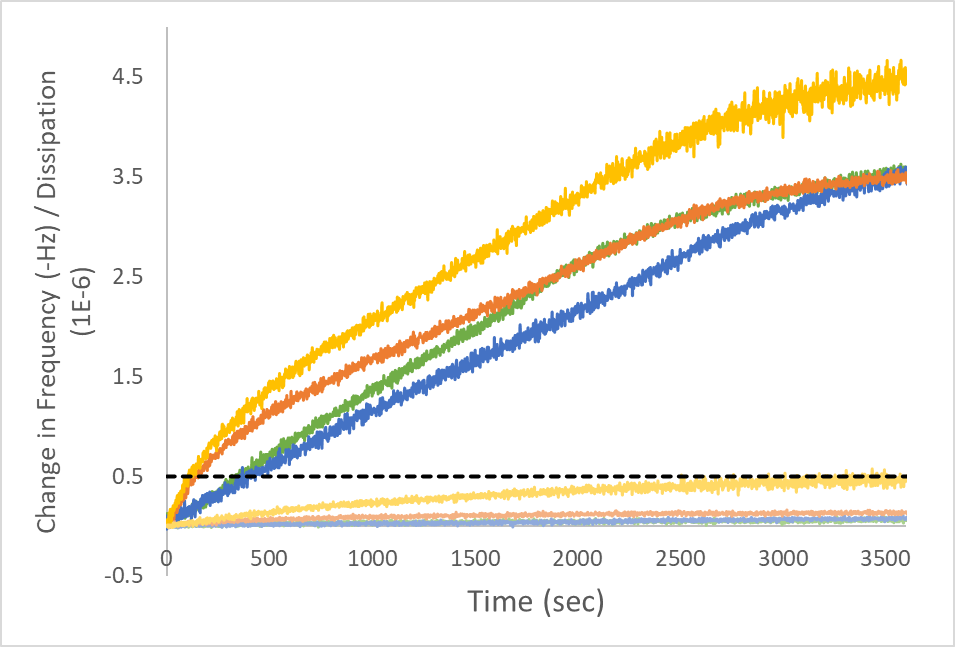


**Supplemental Figure 3.** All frequency and dissipation data collected for 0.1 µM AuBP1. The black dotted line represents the limit of dissipation change that we allowed for this work. The distinct colors represent different experimental runs, while the faded colors represent the dissipation measurement for that corresponding run. The orange curve is the representative curve selected for further analysis.


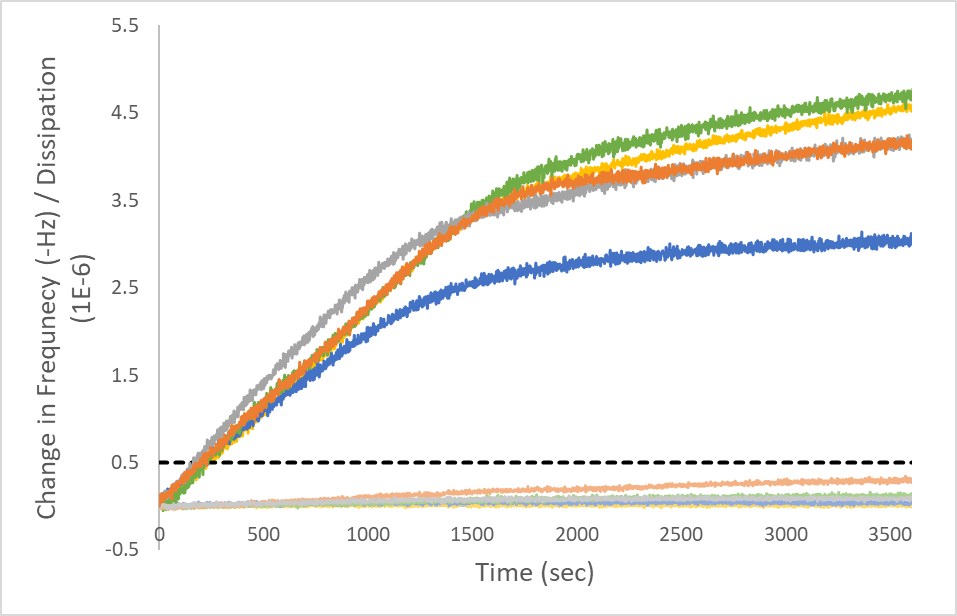


**Supplemental Figure 4.** All frequency and dissipation data collected for 0.15 µM AuBP1. The black dotted line represents the limit of dissipation change that we allowed for this work. The distinct colors represent different experimental runs, while the faded colors represent the dissipation measurement for that corresponding run. The orange curve is the representative curve selected for further analysis.

**
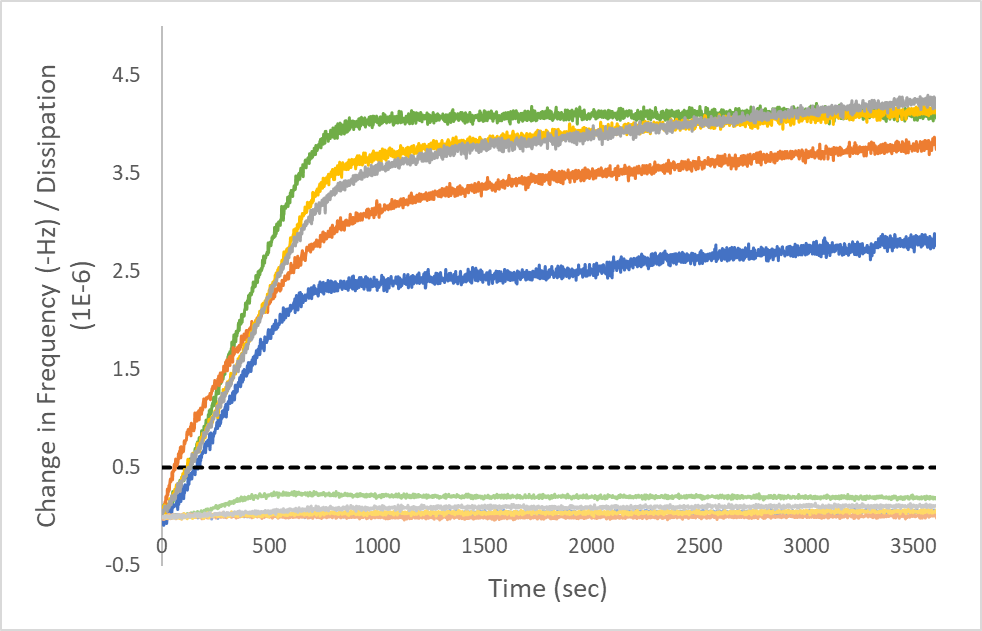
**

**Supplemental Figure 5.** All frequency and dissipation data collected for 0.175 µM AuBP1. The black dotted line represents the limit of dissipation change that we allowed for this work. The distinct colors represent different experimental runs, while the faded colors represent the dissipation measurement for that corresponding run. The orange curve is the representative curve selected for further analysis.

**
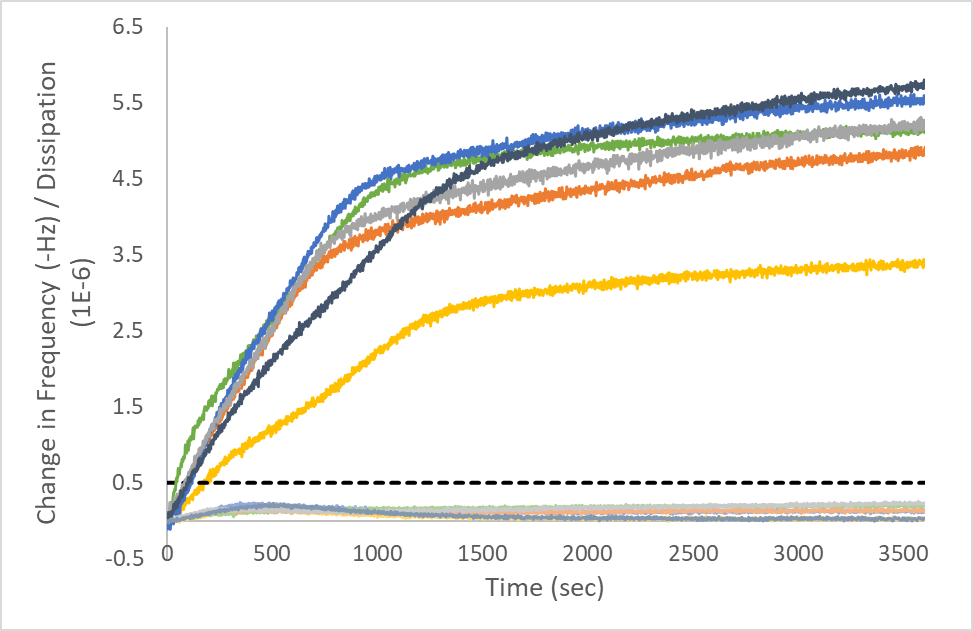
**

**Supplemental Figure 6.** All frequency and dissipation data collected for 0.25 µM AuBP1. The black dotted line represents the limit of dissipation change that we allowed for this work. The distinct colors represent different experimental runs, while the faded colors represent the dissipation measurement for that corresponding run. The orange curve is the representative curve selected for further analysis.

**
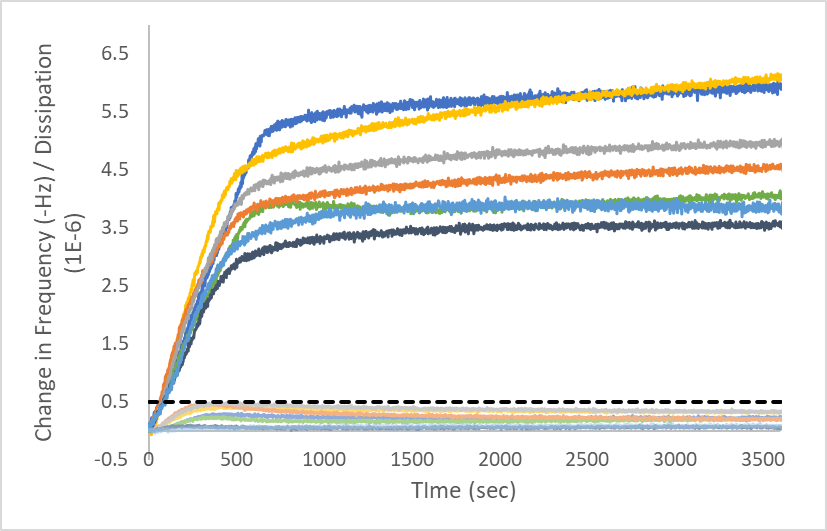
**

**Supplemental Figure 7.** All frequency and dissipation data collected for 0.5 µM AuBP1. The black dotted line represents the limit of dissipation change that we allowed for this work. The distinct colors represent different experimental runs, while the faded colors represent the dissipation measurement for that corresponding run. The orange curve is the representative curve selected for further analysis.

**
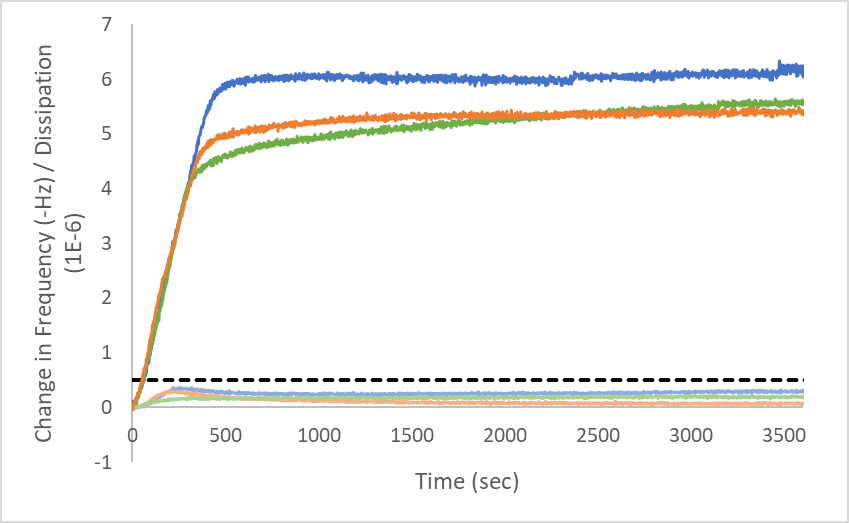
**

**Supplemental Figure 8.** All frequency and dissipation data collected for 0.75 µM AuBP1. The black dotted line represents the limit of dissipation change that we allowed for this work. The distinct colors represent different experimental runs, while the faded colors represent the dissipation measurement for that corresponding run. The orange curve is the representative curve selected for further analysis.

**
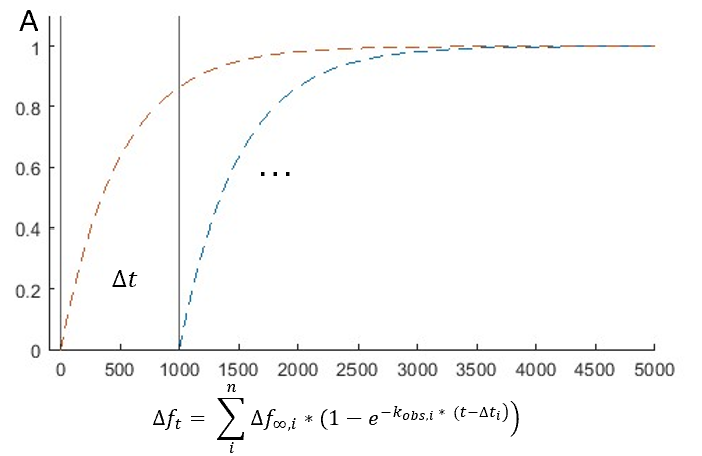
**

**
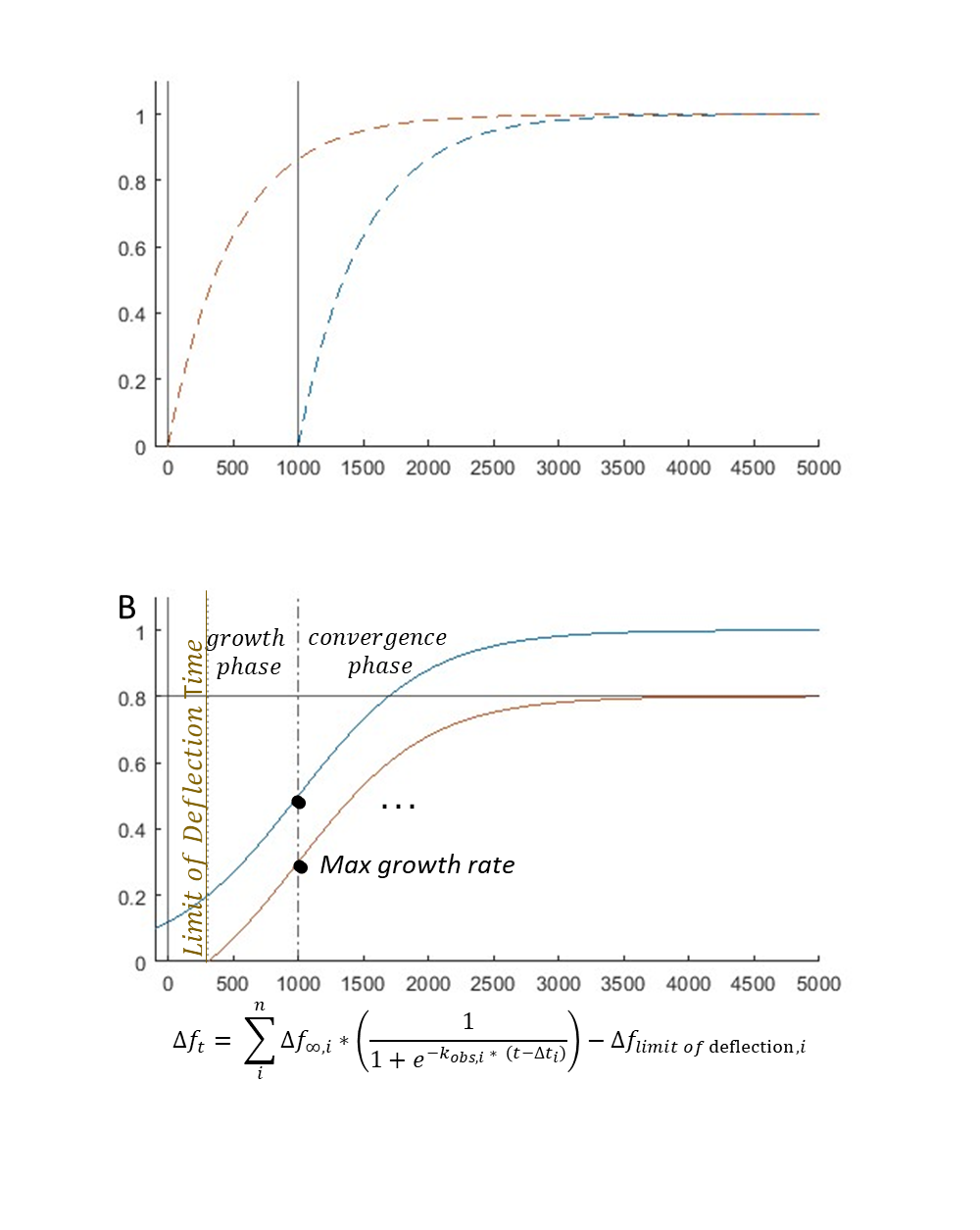
**

**Supplemental Figure 9.** Spectral isotherm descriptions. (A) Time-shifted Langmuir Model, (B) Sigmoidal Curves with changes to the free parameters


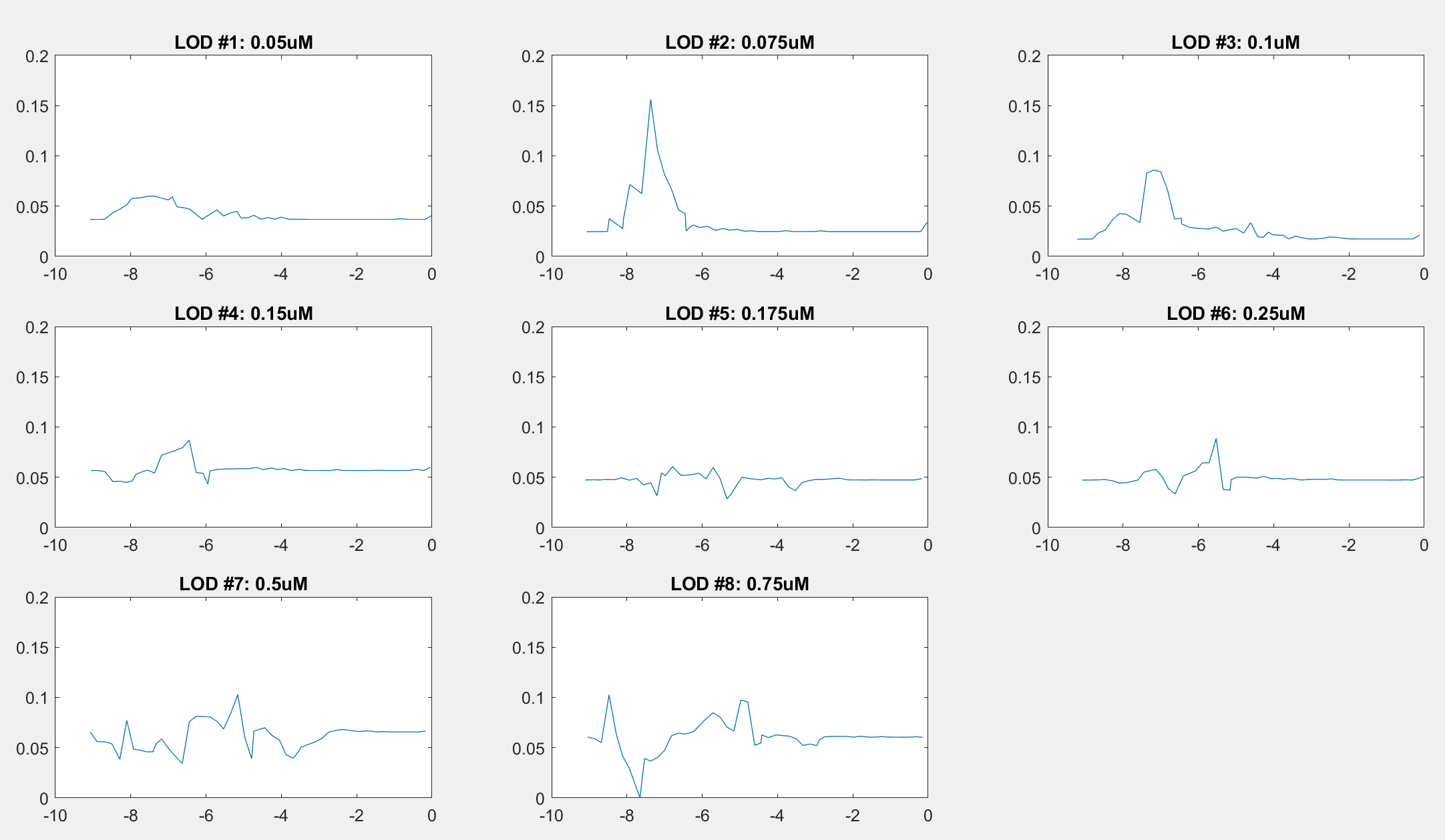


**Supplemental Figure 10.** Limit of Deflection (LOD) as a function of frequency in adsorption measurements

$\ln\frac{Adsorption Hz at 100}{Adsorption Hz at 1 \mu M at 100 sec}=0.96*\ln\left( \left[ AuBP1 \right] \mu M \right)-0.057 (R^{2}=0.82)$

**Supplemental Equation 1.** Derived Hill Equation for the Initial Kinetics of AuBP1 using QCM-D data.


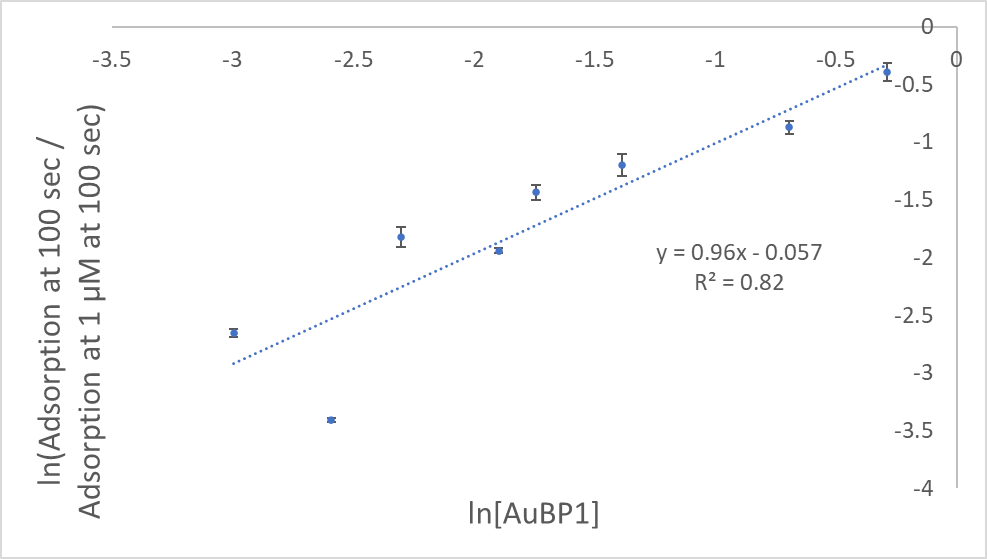


**Supplemental Figure 11.** Hill equation fit for initial kinetics of QCM-D frequency data.


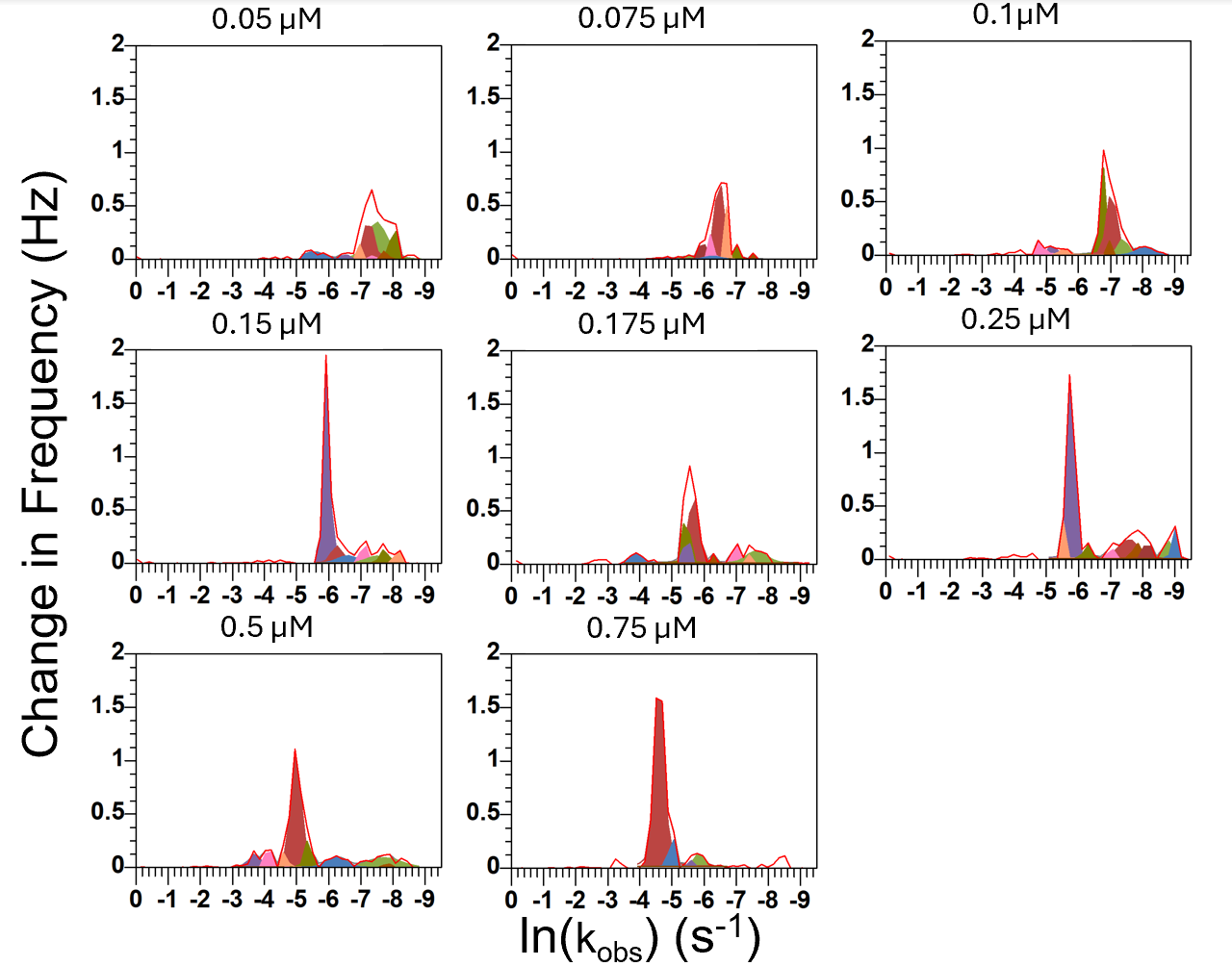


**Supplemental Figure 12.** Peak Deconvolution for all concentrations of AuBP1.
